# Complete Genome Sequence of *Elizabethkingia meningoseptica*, Isolated from a T-Cell Non-Hodgkin’s Lymphoma Patient

**DOI:** 10.1128/genomeA.00673-15

**Published:** 2015-06-25

**Authors:** Guiqin Sun, Lei Wang, Celimuge Bao, Tiansheng Li, Lili Ma, Li Chen

**Affiliations:** a College of Medical Technology, Zhejiang Chinese Medical University, Hangzhou, China; b Key Laboratory of Medical Molecular Virology, School of Basic Medical Sciences, Fudan University, Shanghai, China; c The First Affiliated Hospital of Zhejiang Chinese Medical University, Hangzhou, China

## Abstract

An *Elizabethkingia meningoseptica* infection was detected at the end stage of a patient with T-cell non-Hodgkin’s lymphoma. The complete genome of this isolated strain, FMS-007, was generated in one contig with a total size of 3,938,967 bp. A preliminary screening indicated that the genome contains drug resistance genes to aminoglycosides and β-lactams. A clustered regularly interspaced short palindromic repeats (CRISPR)/CRISPR-associated proteins (CRISPR/Cas) system with 16 direct repeats and 15 spacers was identified.

## GENOME ANNOUNCEMENT

*Elizabethkingia meningoseptica*, also known as *Flavobacterium meningosepticum*, is a nonfermenting, nonmotile, oxidase-positive, Gram-negative bacillus. Studies indicate that clinical *E. meningoseptica* infection may cause meningitis, sepsis, respiratory infection, and other clinical conditions, and the case fatality rate is around 25% (1–3). Although reports regarding *E. meningoseptica*-associated infections are rare, an epidemiological study indicated that the infection is emerging in Taiwan (2), and it is listed in an official survey as one of the top five clinically isolated nonfermenting Gram-negative bacilli in China (4). In this paper, we report the complete genome of *E. meningoseptica* strain FMS-007, a strain isolated from a 15-year-old intensive care unit (ICU) patient with T-cell non-Hodgkin’s lymphoma.

The genomic DNA of FMS-007 was extracted and sequenced with a HiSeq 2000 (Illumina, San Diego, CA) using a whole-genome shotgun strategy. A complete genome of 3,938,967 bp (35.6% G+C content) in one circularized chromosome was generated using SOAPdenovo V1.05 software. No plasmids or phages were detected. Compared to the other reported draft genome sequences of *E. meningoseptica* strains (5–7), the FMS-007 genome is complete and in one contig. With this unique feature, the genome could be used as an ideal template for genomic studies of *E. meningoseptica* and related species.

The gene annotation of the whole genome of FMS-007 was conducted using KEGG, TrEMBL, GO, and COG databases. The results indicated that FMS-007 contains 3,625 putative genes, 5 ribosome RNA operons, and 52 tRNAs. A preliminary screening for drug resistance genes was conducted. An extended-spectrum β-lactamase subtype CME-2, a metalloenzyme subtype BlaB^−^11, GOB-1, and AmpC were identified. Aminoglycoside resistance gene analysis revealed that FMS-007 contained several candidate genes for aminoglycoside resistance, including one 16S rRNA methylase, two aminoglycoside phosphotransferases, and one aminoglycoside nucleotidyltransferase. In addition, a clustered regularly interspaced short palindromic repeats (CRISPR)/CRISPR-associated proteins (CRISPR/Cas) system containing 3 Cas enzymes and a CRISPR cassette with 16 direct repeats and 15 spacers was identified. Current literature indicates that CRISPR enzymes and the CRISPR RNAs may act together as an adaptive immune system to eliminate invaded exogenous DNAs, including phages and/or plasmids (8). The potential role of the detected CRISPR/Cas system in infection and other functions deserves further attention.

### Nucleotide sequence accession number.

This whole-genome shotgun project has been deposited in GenBank under the accession no. CP006576.
